# Anemia Among Hospitalized Children in a Ghanaian Pediatric Emergency Unit: A Prospective Observational Study of Prevalence, Associated Factors, and Hematologic Patterns

**DOI:** 10.1002/hsr2.72505

**Published:** 2026-05-10

**Authors:** Serwah Bonsu Asafo‐Agyei, Emmanuel Ameyaw, Samuel Blay Nguah, Vivian Paintsil, Akua Afriyie Ocran

**Affiliations:** ^1^ School of Medical Sciences Kwame Nkrumah University of Science and Technology Kumasi Ghana; ^2^ Komfo Anokye Teaching Hospital Kumasi Ghana

**Keywords:** anemia, Ghana, hospitalized children, iron deficiency, pediatric emergencies

## Abstract

**Background and Aims:**

Anemia remains a major contributor to pediatric morbidity and mortality in sub‐Saharan Africa, yet contemporary data from tertiary pediatric emergency settings are limited, particularly among children older than 5 years. This study assessed the prevalence, factors associated with anemia, hematological characteristics, and short‐term clinical outcomes among children admitted to a pediatric emergency unit (PEU) in Kumasi, Ghana.

**Methods:**

This prospective observational study enrolled children aged 2 months to 17 years admitted to the PEU of Komfo Anokye Teaching Hospital, Ghana, between November 2024 and January 2025. Sociodemographic, dietary, and clinical data were obtained through caregiver interviews and medical record review. Anemia was defined and graded using age‐ and sex‐specific World Health Organization hemoglobin thresholds. Red cell indices were used to describe anemia morphology. Logistic regression analysis was employed to identify factors independently associated with anemia.

**Results:**

Among 318 children enrolled, 66.7% had anemia. Mild, moderate, and severe anemia accounted for 14.8% (47/318), 34.6% (110/318), and 17.3% (55/318) of cases, respectively. Among children aged 6–59 months, 65.3% (113/173) were anemic. Moderate anemia predominated across age groups, while severe anemia was more frequent among older children. Microcytic anemia with elevated red cell distribution width was the most common morphological pattern, suggesting a high burden of iron deficiency. Anemia was independently associated with clinically presumed iron deficiency, sickle cell disease, and malaria rapid diagnostic test positivity. Nearly one‐quarter of participants required blood transfusion, reflecting the severity of presentations.

**Conclusion:**

Anemia was highly prevalent among children admitted to a tertiary pediatric emergency unit in Ghana and was frequently associated with severe disease requiring blood transfusion. Strengthened strategies focusing on early identification of hemoglobinopathies, prompt malaria control, and improved nutritional assessment and counseling are urgently needed within emergency and inpatient pediatric care.

**Abbreviations**
G6PD: Glucose‐6‐phosphate dehydrogenase
KATH: Komfo Anokye Teaching Hospital
MCV: Mean corpuscular volume
PEU: Pediatric Emergency Unit
PICU: Pediatric Intensive Care Unit
RDT: Rapid diagnostic test
RDW: Red cell distribution width
SCD: Sickle Cell Disease

## 1 Introduction

Anemia, defined as a reduction in red blood cell mass or hemoglobin concentration below normal limits for age and sex, remains a significant global public health challenge [1]. It is both a cause and a consequence of poor health and nutrition and is closely linked to several Sustainable Development Goals, particularly those targeting malnutrition and child survival. Despite sustained global attention, progress in reducing anemia prevalence has been slow, especially in low‐ and middle‐income countries [2, 3].

Iron deficiency remains the leading cause of anemia worldwide, but in children, the etiology is often multifactorial. Nutritional deficiencies, infections such as malaria and helminthiasis, chronic inflammatory conditions, and inherited hemoglobin disorders frequently coexist, particularly in sub‐Saharan Africa. Anemia in childhood is associated with impaired cognitive development, poor school performance, growth retardation, increased susceptibility to infections, heart failure, and mortality [4].

Children under 5 years of age and adolescent girls are considered the most vulnerable groups globally. Worldwide, an estimated 269 million children aged 6–59 months are affected by anemia, with the highest burden in low‐ and lower‐middle‐income countries [1]. In sub‐Saharan Africa, the burden of anemia remains disproportionately high due to the convergence of nutritional inadequacies, infectious diseases, and genetic hemoglobinopathies [5]. Ghana reflects this regional pattern. Although national survey data indicate a gradual decline in anemia prevalence among children aged 6–59 months over the past two decades, substantial disparities persist by age, socioeconomic status, and geographic location [6]. Importantly, national surveys largely capture community prevalence and provide limited insight into clinical severity, morphological patterns, or short‐term outcomes among hospitalized children.

Hospital‐based studies, particularly those conducted in tertiary pediatric emergency units, offer a valuable sentinel perspective on severe and clinically significant anemia. Such settings capture children with acute illness, complex comorbidities, and higher risk of adverse outcomes, including the need for blood transfusion and escalation of care. However, recent prospective data from tertiary emergency settings in West Africa, especially those including children older than 5 years, remain limited. This study, therefore, aimed to determine the prevalence of anemia, describe its morphological patterns, and identify factors associated with anemia among children aged 2 months to 17 years admitted to a tertiary pediatric emergency unit in Ghana.

## 2 Methods

### 2.1 Study Design, Setting, and Period

This prospective observational study was conducted at the Pediatric Emergency Unit (PEU) of Komfo Anokye Teaching Hospital (KATH), a major tertiary referral center in Kumasi, Ghana. The hospital serves as a referral center for 13 out of the 16 regions in Ghana. The PEU at KATH functions as an initial stabilization and short‐stay inpatient unit for acutely ill children. Patients are typically managed for a median duration of 2–3 days before transfer to inpatient wards or the pediatric intensive care unit (PICU), depending on clinical status. Direct discharge from the PEU is uncommon (< 5%). Children with non‐severe conditions are generally managed elsewhere at dedicated outpatient services within the hospital. Consequently, all participants in this study were managed as inpatients. The study was carried out between November 15, 2024, and January 31, 2025. The study duration was determined by the time required to achieve the calculated sample size, and recruitment was done consecutively until the target sample size was reached.

### 2.2 Study Participants

**Figure 1:**
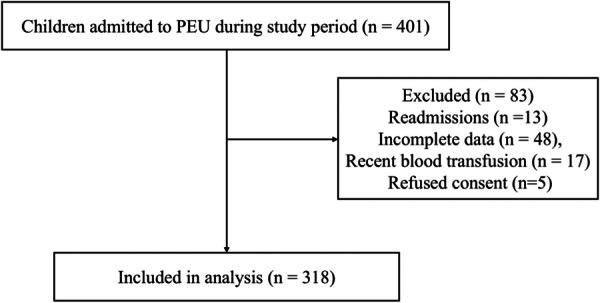
Flow chart of participant recruitment, exclusion and inclusion.

All children aged 2 months to 17 years admitted to the PEU during the study period were eligible. Children were excluded if they were readmissions, had received a blood transfusion within the preceding 3 months, had incomplete data on key anemia‐related variables, or if caregivers declined consent. From a total of 401 children admitted, 318 children were enrolled (Figure 1).

### 2.3 Sample Size Determination

The minimum sample size was calculated using Cochran's formula [7], based on a previously reported anemia prevalence of 28.5% among children admitted to a pediatric emergency unit in a comparable setting [8], a 95% confidence level, and a 5% margin of error. The calculated minimum sample size was 313.

### 2.4 Data Collection Tools, Procedures, and Quality Control

Participants were recruited consecutively by trained research assistants with a background in nutrition, who approached caregivers, explained the study, and obtained consent before enrollment. This was done after the attending medical team had managed any life‐threatening conditions. Data were collected for all participants using a pretested, structured questionnaire administered by research assistants to caregivers, supplemented by a medical records review by the study team. Information obtained included sociodemographic characteristics, dietary practices, breastfeeding history, malaria prevention measures, deworming history, recent blood loss, and clinical diagnoses.

Laboratory tests were performed as part of routine clinical care and were supplemented, when necessary, for study purposes. All participants had hemoglobin concentration and red cell indices measured on admission using an automated hematology analyzer. Additional laboratory investigations, including malaria testing, hemoglobin electrophoresis, peripheral blood film examination, and reticulocyte counts, were performed based on clinical indication.

Venous blood samples were analyzed for hemoglobin concentration and red cell indices using an automated hematology analyzer (Sysmex XN‐2000 Hematology Analyzer; Sysmex, Kobe, Japan). Malaria diagnosis was performed using thick and thin blood films and rapid diagnostic tests. Hemoglobin electrophoresis was conducted for children clinically suspected to have sickle cell disease. Peripheral blood film examination and reticulocyte counts were performed when indicated. Biochemical iron studies were not done in this study. Standard operating protocols and quality control procedures were followed throughout blood specimen collection and analysis, with daily maintenance and calibration of the analyzer machine.

### 2.5 Study Variables and Operational Definitions

Anemia was defined and graded using age‐ and sex‐specific World Health Organization hemoglobin thresholds [9]. For infants under 6 months of age, hemoglobin cutoffs were derived from published reference data (Supplementary Table 1) [10, 11]. Mean corpuscular volume (MCV) was used to classify anemia morphology as microcytic, normocytic, or macrocytic, with red cell distribution width (RDW) categorized as normal or elevated using age‐appropriate reference ranges (Supplementary Table 2) [11, 12]. Hematology analyzer manufacturer reference intervals were not used, as they were not uniformly validated across the full pediatric age range.

Socioeconomic status was classified using the Oyedeji method [13]. Dietary iron intake was assessed in participants aged > 6 months using caregiver‐ or child‐reported food frequency and 24‐h dietary recall, focusing on both heme and non‐heme iron sources. Children who consumed iron‐rich foods at least three times per week were classified as having adequate intake, while those with less frequent consumption were classified as having inadequate intake. Exclusive breastfeeding was defined according to standard age‐appropriate criteria and was assessed for children under 2 years [14]. Deworming within the past 6 months after age 1 year, iron supplementation within 3 months before admission (regardless of indication), and recent blood loss within the past 3 months were documented. Clinical diagnoses were established through standard clinical and laboratory evaluations and coded according to the ICD‐10 classification [15] or the major system involved. Severe acute malnutrition (SAM) was defined as the presence of oedema of both feet (nutritional oedema) or severe wasting (weight‐for‐height/length < −3SD or mid‐upper arm circumference < 115 mm) [16]. Both primary (nutritional) and secondary (from chronic diseases and infections) causes were labelled as Malnutrition.

### 2.6 Clinical Context and Outcomes

Children were admitted for a wide range of acute/chronic medical conditions. While some presented primarily with anemia, others were admitted for conditions unrelated to anemia. A significant proportion of patients referred specifically for anemia had already been hemotransfused before referral and were thus excluded from the study. Clinical outcomes assessed in this study were blood transfusion, length of hospital stay, pediatric intensive care unit admission, and in‐hospital mortality.

### 2.7 Statistical Analysis

The complete data were entered into a Microsoft Excel worksheet previously designed with a data dictionary for the variables to be collected during the study. It was consistently checked for any incorrect, duplicated or missing data, which were corrected immediately.

Data were analyzed using R version 4.5.2. Continuous variables were assessed for normality and summarized as means (SD) or medians (IQR), as appropriate. Categorical variables were summarized as frequencies and percentages. Anemia was defined and graded based on age‐ and sex‐specific WHO hemoglobin thresholds. Red cell indices were categorized to describe the type of anemia, with MCV classified as microcytic, normocytic, or macrocytic, and RDW as normal or elevated. Cross‐tabulations of MCV and RDW were used to identify morphological patterns. For the initial bivariate analysis, comparisons between children with and without anemia were conducted using the Pearson *χ*² test or Fisher's exact test for categorical variables, and the Wilcoxon rank‐sum test for continuous variables. There were fewer than 2% missing observations, so all analyses were conducted with complete cases.

Univariate logistic regression was used to identify factors associated with anemia, and effect sizes were presented as crude odds ratios (CORs) with 95% confidence intervals. Variables with *p *< 0.10 in the univariate analysis were included in a multivariable logistic regression to estimate adjusted odds ratios (AORs) with 95% confidence intervals. Furthermore, a univariate ordinal regression was used to assess the association between clinical covariates and increasing anemia severity. Multicollinearity checks were performed prior to model fitting, and all model diagnostics were conducted to ensure validity.

All tests were two‐sided, and *p*‐values < 0.05 were considered statistically significant. The analyses performed in this study were pre‐specified in accordance with the study objectives and analysis plan.

### 2.8 Ethical Considerations

The study adhered to the ethical principles of the Declaration of Helsinki. Ethical approval was obtained from the KATH Institutional Review Board (KATH IRB/AP/178/24). Written informed consent was obtained from parents or caregivers before enrollment.

## 3 Results

A total of 318 children were enrolled, of whom 66.7% had anemia. Among children aged 6–59 months, 65.3% were anemic. The median hemoglobin concentration was 9.8 g/dL (IQR 8−11.4). Inadequate dietary iron intake was common, particularly among anemic children.

Anemia was more frequent among children from lower socioeconomic backgrounds and those with a history of recent blood loss. Clinically presumed iron deficiency, sickle cell disease, and malaria RDT positivity were markedly more common among anemic children. Anemia was associated with longer hospital stays and a higher likelihood of requiring blood transfusion, while rates of PICU admission and in‐hospital mortality were similar between anemic and non‐anemic groups (Table 1).

**Table 1:** Demographic and clinical characteristics of study participants by Anemia status.

	Anemia			
Characteristic	No *N* = 106^†^	Yes *N* = 212^†^	Overall *N* = 318^†^	*p* value^‡^
**Socio‐demographic factors**				
Age (Months)	23 (10, 68)	47 (16, 106)	38 (14, 93)	**< 0.001**
Sex				0.63
Female	50 (47.2%)	94 (44.3%)	144 (45.3%)	
Male	56 (52.8%)	118 (55.7%)	174 (54.7%)	
Residence type				0.29
Rural	38 (35.8%)	89 (42.0%)	127 (39.9%)	
Urban	68 (64.2%)	123 (58.0%)	191 (60.1%)	
Mother's educational level				0.65
None	10 (9.4%)	22 (10.4%)	32 (10.1%)	
Primary	41 (38.7%)	89 (42.0%)	130 (40.9%)	
Secondary	33 (31.1%)	69 (32.5%)	102 (32.1%)	
Tertiary	22 (20.8%)	32 (15.1%)	54 (17.0%)	
Family socioeconomic status				**0.02**
Low	62 (58.5%)	155 (73.1%)	217 (68.2%)	
Middle	40 (37.7%)	48 (22.6%)	88 (27.7%)	
High	4 (3.8%)	9 (4.2%)	13 (4.1%)	
**Dietary factors**				
Exclusive breastfeeding	47 (66.2%)	59 (54.6%)	106 (59.2%)	0.12
Adequate dietary iron	62 (68.9%)	104 (51.2%)	166 (56.7%)	**0.005**
Iron supplementation	49 (46.2%)	76 (35.8%)	125 (39.3%)	0.07
**Preventive health practices**				
Malaria prevention method	82 (77.4%)	161 (75.9%)	243 (76.4%)	0.78
Dewormed (past 6 months)	47 (57.3%)	101 (56.1%)	148 (56.5%)	0.86
**Clinical and disease‐related factors**				
Recent blood Loss	6 (5.7%)	31 (14.6%)	37 (11.6%)	**0.02**
Sickle cell disease	2 (1.9%)	41 (19.3%)	43 (13.5%)	**< 0.001**
G6PD deficiency	0 (0.0%)	10 (4.7%)	10 (3.1%)	**0.03**
Iron deficiency	3 (2.8%)	94 (44.3%)	97 (30.5%)	**< 0.001**
Malignancy	5 (4.7%)	14 (6.6%)	19 (6.0%)	0.50
Malaria (RDT positive)	3 (2.8%)	41 (19.3%)	44 (13.8%)	**< 0.001**
Pneumonia	5 (4.7%)	26 (12.3%)	31 (9.7%)	**0.03**
Sepsis	7 (6.6%)	33 (15.6%)	40 (12.6%)	**0.02**
Malnutrition	15 (14.2%)	34 (16.0%)	49 (15.4%)	0.66
**Clinical outcomes**				
PICU admission	3 (2.8%)	10 (4.7%)	13 (4.1%)	0.56
Blood transfusion	3 (2.8%)	73 (34.4%)	76 (23.9%)	**< 0.001**
Duration of hospital stay	5 (2, 8)	6 (3, 11)	6 (3, 10)	**0.04**
In‐hospital mortality	9 (8.5%)	21 (9.9%)	30 (9.4%)	0.68

*Note:* ^†^Median (Q1, Q3); *n* (%); ^‡^Wilcoxon rank sum test; Pearson's Chi‐squared test; Fisher's exact test; Bold values denote statistically significant results (*p* < 0.05).

### 3.1 Severity of Anemia by Age and Gender

Anemia severity varied by age and sex. Moderate anemia was the most prevalent category across all age groups. Severe anemia occurred more frequently among school‐aged children and adolescents compared with younger children. Overall, boys aged between 12 and 14 years had the highest prevalence of anemia (10/12; 83.3%) followed by children aged 5–11 years (67/90; 74.4%) [Table 2].

**Table 2:** Anemia severity by age and gender.

	Anemia severity				
	None	Mild	Moderate	Severe	Total
**Categorized age**					
2 to 5 months	16 (64.0%)	4 (16.0%)	2 (8.0%)	3 (12.0%)	25
6 to 23 months	37 (38.9%)	22 (23.2%)	29 (30.5%)	7 (7.4%)	95
24 to 59 months	23 (29.5%)	14 (17.9%)	33 (42.3%)	8 (10.3%)	78
5 to 11 years	23 (25.6%)	4 (4.4%)	33 (36.7%)	30 (33.3%)	90
12 to 14 years (girls)	5 (35.7%)	2 (14.3%)	3 (21.4%)	4 (28.6%)	14
12 to 14 years (boys)	2 (16.7%)	1 (8.3%)	7 (58.3%)	2 (16.7%)	12
≥ 15 years (girls)	0 (0.0%)	0 (0.0%)	3 (75.0%)	1 (25.0%)	4
**Total**	106 (33.3%)	47 (14.8%)	110 (34.6%)	55 (17.3%)	318

### 3.2 Red Cell Indices

Microcytic anemia was the predominant morphological pattern, accounting for the majority of anemic cases. Elevated RDW was significantly more common among children with anemia. (Table 3A). Cross‐tabulation of MCV and RDW showed that microcytic–high RDW patterns were the most common, particularly among children with anemia (Table 3B).

**Table 3A:** Red cell indices stratified by anemia status.

	Anemia present			
Characteristic	No *N* = 106^†^	Yes *N* = 212^†^	Overall *N* = 318^†^	*p* value^‡^
**Categorized RDW**				**< 0.001**
Normal	53 (50.0%)	40 (18.9%)	93 (29.2%)	
High	53 (50.0%)	172 (81.1%)	225 (70.8%)	
**Categorized MCV**				0.180
Microcytic	67 (63.2%)	128 (60.4%)	195 (61.3%)	
Normocytic	38 (35.8%)	73 (34.4%)	111 (34.9%)	
Macrocytic	1 (0.9%)	11 (5.2%)	12 (3.8%)	

*Note:* ^†^ *n* (%); ^‡^Pearson's Chi‐squared test; Fisher's exact test; Bold values denote statistically significant results (*p* < 0.05).

**Table 3B:** Cross tabulation of MCV with RDW.

	Anemia absent			Anemia Present		
RDW	Normal	High	Total	Normal	High	Total
**Categorized MCV**						
Microcytic	27 (25%)	40 (38%)	67 (63%)	20 (9.4%)	108 (51%)	128 (60%)
Normocytic	26 (25%)	12 (11%)	38 (36%)	18 (8.5%)	55 (26%)	73 (34%)
Macrocytic	0 (0%)	1 (0.9%)	1 (0.9%)	2 (0.9%)	9 (4.2%)	11 (5.2%)
**Total**	53 (50%)	53 (50%)	106 (100%)	40 (19%)	172 (81%)	212 (100%)

### 3.3 Factors Associated With Anemia

Table 4 shows factors associated with anemia in univariate and multivariable logistic regression analyses. Adjusted odds ratios (AOR) represent associations after controlling for confounders. In the multivariate analysis, anemia was independently associated with clinically presumed iron deficiency, sickle cell disease, malaria rapid diagnostic test positivity and blood transfusion.

**Table 4:** Univariate and Multivariable logistic regression analysis showing independent factors associated with Anemia.

	Univariate				Multivariate		
Variable	*N*	COR	95% CI	*p* value	AOR	95% CI	*p* value
Age (months)	318	1.01	1.00, 1.01	**0.002**	1.00	1.00, 1.01	0.22
Sex, male	318	1.12	0.70, 1.79	0.63			
Residence type	318						
Rural		—	—				
Urban		0.77	0.47, 1.25	0.29			
Mother's educational level	318						
None		—	—				
Primary		0.99	0.41, 2.23	0.98			
Secondary		0.95	0.39, 2.20	0.91			
Tertiary		0.66	0.26, 1.64	0.38			
Family socioeconomic status	318						
Low		—	—		—	—	
Middle		0.48	0.29, 0.80	**0.005**	0.90	0.43, 1.91	0.79
High		0.90	0.28, 3.42	0.87	1.27	0.26, 6.24	0.77
Exclusive breastfeeding	179	0.61	0.33, 1.14	0.13			
Adequate dietary iron	293	0.47	0.28, 0.80	**0.005**	0.74	0.34, 1.63	0.46
Iron supplementation	318	0.65	0.40, 1.04	0.08	0.77	0.38, 1.55	0.47
Malaria prevention method	318	0.92	0.52, 1.59	0.78			
Dewormed (past 6 months)	262	0.95	0.56, 1.61	0.86			
Blood loss (past 3 months)	318	2.85	1.23, 7.80	**0.02**	1.65	0.53, 5.49	0.39
Malnutrition	318	1.16	0.61, 2.29	0.67			
Iron deficiency	318	25.8	9.32, 107	**< 0.001**	**37.7**	10.7, 241	**< 0.001**
Sickle Cell Disease	318	12.5	3.73, 77.5	**< 0.001**	**10.5**	2.57, 71.5	**0.004**
Sepsis	318	2.61	1.18, 6.62	**0.03**	**2.30**	0.65, 9.03	0.21
Malignancy	318	1.43	0.53, 4.52	0.50			
Malaria (RDT positive)	318	8.23	2.90, 34.6	**< 0.001**	**7.65**	2.29, 35.2	**0.003**
Pneumonia	318	2.82	1.14, 8.55	**0.04**	1.74	0.47, 6.68	0.40
PICU admission	318	1.70	0.51, 7.70	0.43			
Blood transfusion	318	18.0	6.48, 75.0	**< 0.001**	**12.0**	3.18, 79.1	**0.001**
Duration of hospital stay	318	1.03	1.00, 1.07	**0.03**	1.02	0.97, 1.07	0.43
In‐hospital mortality	318	1.18	0.54, 2.82	0.68			

Abbreviations: AOR, Adjusted Odds Ratio; CI, Confidence Interval, COR, Crude Odds Ratio.

Bold values denote statistically significant results (*p* < 0.05).

All patients with G6PD deficiency (*n* = 10) were anemic; therefore, this variable was excluded from the logistic regression analysis due to complete separation. Other factors, including socioeconomic status, dietary iron intake, infection, and duration of hospital stay, showed associations in univariate analysis but did not retain independent significance after adjustment (Table 4).

Anemia severity was further examined to assess its influence on clinical management and associated diagnoses. Increasing severity was significantly associated with higher odds of blood transfusion and longer hospital stay. Additionally, severe anemia was significantly associated with malaria, sickle cell disease, G6PD deficiency, iron deficiency and pneumonia. The other covariates were not crudely associated with worsening anemia severity. (Table 5).

**Table 5:** Ordinal regression analysis of the association between anemia severity, clinical diagnoses, and patient outcomes.

	Anemia category							
Characteristic	None *N* = 106^*a*^	Mild *N* = 47^*a*^	Moderate *N* = 110^*a*^	Severe *N* = 55^*a*^	Overall *N* = 318^*a*^	OR	95% CI	*p* value
**Underlying diagnoses**								
Malnutrition	15 (14%)	6 (13%)	19 (17%)	9 (16%)	49 (15%)	1.19	0.68, 2.07	0.54
Iron deficiency	3 (2.8%)	17 (36%)	49 (45%)	28 (51%)	97 (31%)	5.14	3.28, 8.18	**< 0.001**
Sickle cell disease	2 (1.9%)	0 (0%)	21 (19%)	20 (36%)	43 (14%)	7.96	4.28, 15.1	**< 0.001**
G6PD deficiency	0 (0%)	1 (2.1%)	6 (5.5%)	3 (5.5%)	10 (3.1%)	3.48	1.19, 10.6	**0.02**
Sepsis	7 (6.6%)	9 (19%)	19 (17%)	5 (9.1%)	40 (13%)	1.36	0.77, 2.40	0.29
Malignancy	5 (4.7%)	2 (4.3%)	6 (5.5%)	6 (11%)	19 (6.0%)	1.85	0.77, 4.48	0.17
Malaria (RDT positive)	3 (2.8%)	6 (13%)	21 (19%)	14 (25%)	44 (14%)	3.52	1.99, 6.32	**< 0.001**
Pneumonia	5 (4.7%)	3 (6.4%)	11 (10%)	12 (22%)	31 (9.7%)	3.26	1.63, 6.63	**< 0.001**
**Clinical outcomes**								
PICU admission	3 (2.8%)	5 (11%)	4 (3.6%)	1 (1.8%)	13 (4.1%)	0.82	0.32, 2.09	0.68
Blood transfusion	4 (3.8%)	3 (6.4%)	9 (8.2%)	9 (16%)	25 (7.9%)	2.82	1.33, 6.11	**0.007**
Duration of hospital stay	5 (2, 8)	5 (2, 7)	6 (3, 11)	8 (4, 14)	6 (3, 10)	1.04	1.02, 1.07	**< 0.001**
In‐hospital mortality	9 (8.5%)	5 (11%)	12 (11%)	4 (7.3%)	30 (9.4%)	1.01	0.52, 1.96	0.98

*Note:* ^*a*^ *n* (%); Median (Q1, Q3).

Abbreviations: CI, confidence interval; OR, odds ratio.

Bold values denote statistically significant results (*p* < 0.05).

**Figure 2:**
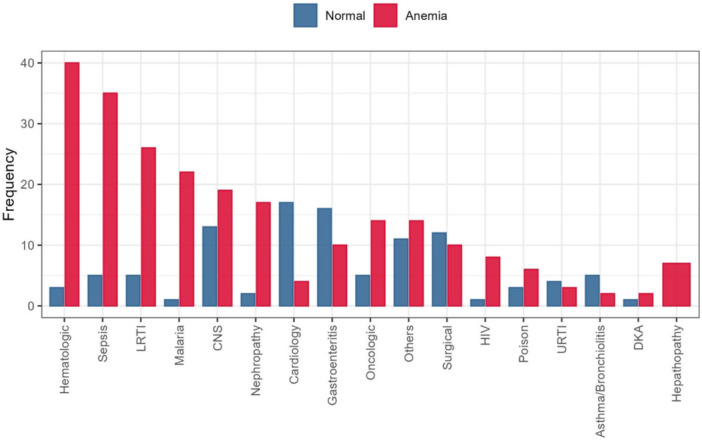
Discharge diagnoses stratified by anemia status among study participants.

Figure 2 further shows the distribution of discharge diagnoses based on the major system involved or ICD‐10 code [15] stratified by anemia status.

## 4 Discussion

This study demonstrates a high prevalence of anemia among children admitted to a tertiary pediatric emergency unit in Ghana, affecting two‐thirds of participants. The burden was substantial across all age groups and particularly high among older children and adolescents, a population often underrepresented in anemia research. Our prevalence exceeds many hospital‐based reports from similar settings but falls within the globally reported ranges (19.3%–83.2%) [17, 18, 19, 20, 21, 22, 23, 24, 25]. The wide range likely reflects differences in participants' socio‐demographic characteristics, disease burden and severity and diet. Our rate is higher than most studies of hospitalized children that included those over 5 years, exceeding the reported 28.5% in Sudan [8], 34.8% in Nigeria [23], and 58.6% in Ethiopia [17]. Rates are much lower in high‐income settings; Cusick et al. [24] reported a rate of 19.3% in an urban community hospital in the United States.

Globally, about 40% of children aged 6–59 months are reported to be anemic with higher rates in low‐ and middle‐income countries [1]. Our study's prevalence rate of 65.3% among under‐fives was higher than other hospital‐based estimates, including 55% in another Ghanaian hospital [18] and 57.2% reported in Ethiopia [19], similar to 63.3% in India [25] and lower than 83.2% in Tanzania [22]. As expected, hospital prevalence exceeded community estimates, including 48.9% in the latest Ghana Demographic and Health Survey [26] and 54% reported by Aheto et al. [27], reflecting referral bias and the concentration of severely ill children in tertiary emergency settings.

Unlike studies that showed the highest anemia burden in ages 6–59 months [23, 28], we found a relatively higher prevalence among older children, similar to a report from Ethiopia [17]. This may reflect school‐related dietary patterns, with reliance on iron‐poor, carbohydrate‐rich vendor foods. Although the national school feeding program was introduced to mitigate this risk, the implementation has been challenging [29].

Moderate anemia was the most common severity category, while severe anemia accounted for a significant proportion of cases, reflecting the acuity and complexity of presentations in emergency settings. The predominance of microcytic anemia with elevated RDW strongly suggests iron deficiency as the leading contributor, aligning with global estimates [1]; however, overlapping etiologies, including malaria infection and hemoglobinopathies are likely contributory in this setting. Mild to moderate anemia and red cell indices abnormalities may be overlooked in clinical practice, with inadequate discharge management. Appropriate nutritional counselling and iron supplementation at discharge could be an important step toward reducing anemia burden and improving outcomes. Reducing anemia, particularly in children, is highly cost‐effective and associated with substantial health benefits [30]. Exclusive breastfeeding was not significantly associated with anemia, though fewer anemic children had been exclusively breastfed. While breast milk provides sufficient bioavailable iron in early infancy [31], delayed or inadequate complementary feeding beyond 6 months may negate this protective effect [32, 33]. In our study, reliance on cereal‐based diets with low iron bioavailability may have negated the early protective effects of exclusive breastfeeding. Nutritional counseling should therefore emphasize both optimal breastfeeding practices and timely introduction of iron‐rich complementary foods.

Deworming had no significant association with anemia in our study. This could be due to the frequent mass deworming among school‐aged children in Ghana and the overall considerable prevalence of deworming in the study population, likely leading to a decreased burden of parasitic diseases. Other studies reported a negative correlation between deworming and anemia [34, 35]. Malnutrition also showed no significant association, possibly due to the inclusion of secondary malnutrition from causes such as cyanotic congenital heart disease.

Iron deficiency, sickle cell disease, and malaria infection were independently associated with anemia. These findings reflect the well‐recognized interplay between nutritional deficiencies, inherited hemoglobinopathies, and infectious diseases in sub‐Saharan Africa [5]. The strong association with sickle cell disease underscores the importance of early diagnosis and comprehensive care for affected children. Malaria remains a significant contributor through hemolysis and impaired erythropoiesis [36], reinforcing the need for sustained malaria prevention and prompt treatment.

Further stratification by anemia severity demonstrated differences in both underlying diagnoses and clinical management. Increasing anemia severity was significantly associated with higher odds of having malaria, sickle cell disease, G6PD deficiency, iron deficiency, and pneumonia; buttressing that a combination of infectious, genetic, and nutritional factors largely drives severe anemia in this setting. The severity of anemia also influenced clinical management, with children who had severe anemia being more likely to receive blood transfusions, whereas those with milder anemia were usually managed with hematinics and dietary counseling. Nearly one‐quarter of children required blood transfusion, highlighting the severity of anemia encountered in tertiary emergency care and the associated strain on health care resources. Anemic children also experienced longer hospital stays, further increasing the economic and social burden on families and health systems. Although mortality was higher among anemic children, the difference was not statistically significant, supporting evidence that anemia often reflects underlying disease severity rather than acting as an isolated cause of death [37].

### 4.1 Strengths and Limitations

The strengths of this study include its prospective design, inclusion of a wide pediatric age range, and focus on a tertiary emergency setting, providing clinically relevant insights into severe anemia. Limitations include the single‐center design, short study duration, and lack of biochemical iron studies, which limited definitive classification of iron deficiency. Referral bias inherent to tertiary emergency settings is also a limitation.

### 4.2 Conclusions and Recommendations

Anemia is a highly prevalent and clinically significant comorbidity among children presenting for emergency care in low‐resource settings. Integrated strategies incorporating routine hemoglobin screening, early identification of hemoglobinopathies, strengthened malaria control, and structured nutritional assessment and counseling should be embedded within pediatric emergency and inpatient care pathways to reduce the burden and consequences of childhood anemia.

## Author Contributions

**Serwah Bonsu Asafo‐Agyei:** conceptualization, investigation, writing – original draft, methodology, data curation, supervision, project administration, writing – review and editing, validation. **Emmanuel Ameyaw:** conceptualization, data curation, investigation, methodology, writing – review and editing, validation. **Samuel Blay Nguah:** data curation, formal analysis, software, writing – review and editing, methodology, validation. **Vivian Paintsil:** data curation, investigation, methodology, writing – review and editing. **Akua Afriyie Ocran:** data curation, investigation, methodology, writing – review and editing, validation.

## Funding

The authors have nothing to report.

## Conflicts of Interest

The authors declare no conflicts of interest.

## Transparency Statement

The lead author Serwah Bonsu Asafo‐Agyei affirms that this manuscript is an honest, accurate, and transparent account of the study being reported; that no important aspects of the study have been omitted; and that any discrepancies from the study as planned (and, if relevant, registered) have been explained.

## Supporting information


**Table S1:** Age‐specific hemoglobin cutoffs used to define anemia severity in this study [9‐11].

**Table S2:** Age‐specific reference ranges for MCV and RDW used in this study [11, 12].

## Data Availability

The data that support the findings of this study are available from the corresponding author upon reasonable request.
