# Supplementary material for: Anemia Among Hospitalized Children in a Ghanaian Pediatric Emergency Unit: A Prospective Observational Study of Prevalence, Associated Factors, and Hematologic Patterns
Source: Health Sci Rep. 2026 May 10;9(5):e72505. doi: 10.1002/hsr2.72505 (PMC13158158; doi:10.1002/hsr2.72505)
Supplement: Supplementary file 1 — Table S1: Age‐specific hemoglobin cutoffs used to define anemia severity in this study [9‐11]. [file HSR2-9-e72505-s002.docx]

**Supplementary Table 1: Age-specific hemoglobin cutoffs used to define anemia severity in this study [9-11]**

| Population | No anemia | Mild anemia | Moderate anemia | Severe anemia |
| --- | --- | --- | --- | --- |
| Children, 2 – 5 months | ≥95 | 80–94 | 70–79 | <70 |
| Children, 6–23 months | ≥105 | 95–104 | 70–94 | <70 |
| Children, 24–59 months | ≥110 | 100–109 | 70–99 | <70 |
| Children, 5–11 years | ≥115 | 110–114 | 80–109 | <80 |
| Children, 12–14 years, nonpregnant girls | ≥120 | 110–119 | 80–109 | <80 |
| Children, 12–14 years, boys | ≥120 | 110–119 | 80–109 | <80 |
| Adults, 15–65 years, nonpregnant women | ≥120 | 110–119 | 80–109 | <80 |
| Adults, 15–65 years, men | ≥130 | 110–129 | 80–109 | <80 |
