# Supplementary material for: Anemia Among Hospitalized Children in a Ghanaian Pediatric Emergency Unit: A Prospective Observational Study of Prevalence, Associated Factors, and Hematologic Patterns
Source: Health Sci Rep. 2026 May 10;9(5):e72505. doi: 10.1002/hsr2.72505 (PMC13158158; doi:10.1002/hsr2.72505)
Supplement: Supplementary file 2 — Table S2: Age‐specific reference ranges for MCV and RDW used in this study [11, 12]. [file HSR2-9-e72505-s001.docx]

**Supplementary Table 2: Age-specific reference ranges for MCV and RDW used in this study [11, 12]**

| Age | MCV (fL) | | RDW (%) | |
| --- | --- | --- | --- | --- |
|  | Lower limit  2.5^th^ percentile | Upper limit  97.5^th^ percentile | Lower limit | Upper limit |
| 2 – 5 months | 74 | 108 | 11.0 | 16.5 |
| 6 months to <2 years | 73 | 85 | 12.3 | 15.6 |
| 2 to 6 years | 75 | 86 | 12.0 | 14.6 |
| 6 to 12 years | 78 | 90 | 11.9 | 13.8 |
| Female >12 years | 80 | 96 | 11.9 | 14.6 |
| Male > 12 years | 80 | 96 | 11.9 | 13.7 |
